# The high costs facing TB-affected households in Mali

**DOI:** 10.5588/ijtld.22.0290

**Published:** 2022-11-01

**Authors:** M. Traore, P. Nguhiu, N. Telly, S. Traore, Y. Toloba, F. Camara, M. Keita, B. Konaté, Y. Diallo, Z. Diallo, N. Bah, M. Dieffaga, S. Laokri, I. Garcia Baena

**Affiliations:** 1 Ministère de la Santé et du Développement Social, Cellule Sectorielle de Lutte contre le VIH/Sida, la Tuberculose et les Hépatites Virales, Bamako, Mali; 2 World Health Organization, Global TB Programme, Geneva, Switzerland; 3 Ministère de la Santé et du Développement Social, Centre Hospitalier Universitaire du Point G, Bamako, Mali; 4 Ministère de la Santé et du Développement Social, Institut Nationale de Santé Publique/Laboratoire Nationale de Référence, Bamako, Mali; 5 World Health Organization, Bamako, Mali; 6 Ministère de la Santé et du Développement Social, Direction Nationale du Développement Social, Bamako, Mali; 7 World Health Organization, Global TB Programme Technical Assistance, Geneva, Switzerland; 8 Tulane University, School of Public Health and Tropical Medicine, New Orleans, LA, USA

Dear Editor,

The Global End TB Strategy recommends that countries monitor not only the mortality and incidence of TB, but also the economic burden of TB on affected households.^1^ The WHO has provided guidance for countries needing to monitor this indicator through household surveys.^2^ By March 2022, over 25 countries had implemented a survey and provided evidence previously unavailable to benchmark and monitor the proportion of TB-affected households that experienced costs above 20% of their annual household income.^3^ With a percapita nominal GDP of US$862 in 2020 and a poverty headcount of 43.8%, Mali spent US$34 per capita on healthcare in 2019, comprising 43% from the government and 31% from households.^4^ In 2020, 7,141 cases were notified and treated free-of-charge at 83 high-level public health facilities (fee-for-services included diagnosis and treatment of TB-related adverse events, radiology and fine-needle biopsies). In 2020, the WHO estimates that delivering TB prevention, diagnosis and treatment in Mali cost respectively US$596 and US$11,673 per drug-susceptible (DS-) and drug-resistant (DR-) TB patient.^3^ In 2021, to cover these costs, Mali committed US$6.1 million of the US$6.8 million required to achieve the national TB plan (NTP) targets, 39% of which was expected to come from domestic resources. The country still faced a funding gap of US$0.7 million.

We have conducted a national, cross-sectional, facility-based survey to characterise the costs faced by TB-affected households seeking care in health facilities within the NTP network, in line with WHO guidance.^2^ Thirty-one TB treatment facilities were sampled at random, with selection probability proportional to cases notified in 2019, including one facility that focuses on treatment of DR-TB. Due to security concerns, facilities in Bankas District and Koro District were replaced with those in Kati and those in Yanfolila, respectively. All adults and children on TB treatment in the selected facilities for 14 or more days in either intensive or continuation phase between 7 July and 31 August 2021 were eligible for consecutive sampling. The study protocol was approved by the *Comité national d’éthique pour la santé et les sciences de la vie*, Bamako, Mali (107/MSAS/CNESS) and WHO African Region Ethics Review Board (AFR/ERC/2021/2.5). Written informed consent was obtained from adults and children’s caregivers prior to each interview. Thirteen trained interviewers administered standardised electronic questionnaires in French, deployed through the ONA^®^ platform (https://ona.io/home/about/mission-vision). Patients’ history of health service utilisation in relation to TB care, medical and non-medical costs, time lost in seeking treatment, household income and asset ownership and household-level impact and coping mechanisms, including changes in self-reported income were collected. Financial data were reported in West African francs (CFA) and converted using US$1 = CFA 537.3 based on the prevailing exchange rate on 1 June 2021.

Of the 453 patients who participated in the survey (439 DS-TB, 14 DR-TB), 12% were unemployed, which is higher than the national rate (1.6%).^5^ Only 15 respondents (3.3%) reported receiving any form of social protection, although two-thirds of the survey respondents were not the main income earners, and the annual household income averaged US$4,589. In 2021, the total TB episode costs incurred by affected households averaged US$1,087 (95% confidence interval [CI] 895–1,279) (Table). The proportion of TB-affected households experiencing costs higher than 20% of their annual household income as per WHO global monitoring indicator^1^ was 49.4% (95% CI 40.7–58.2) for all patients. As a result of these costs, the number of TB-affected households estimated below the international poverty line would increase from 26% (95% CI 20–33) to 54% (95% CI 46–61). The main risk factors associated with catastrophic costs in this survey were hospitalisation (adjusted odds ratio [aOR] 5.7, 95% CI 2.6–12.2) and household income quintile, with the lowest quintile having 40 times higher odds than the wealthiest quintile (aOR 40.4, 95% CI 15.8–103.7). Although most patients in Mali (55%) were informally employed at onset of TB, there was a huge impact on patients’ ability to work: 82% of respondents reported that TB diagnosis led to lost days of work and 42% reported job losses. Further social effects reported included food insecurity (18%) and social exclusion (13%). Almost all patients (81%) felt that they had become poorer or much poorer after TB diagnosis, and 60% reported having to either borrow funds or sell assets to alleviate the economic burden imposed by TB. These and further results are available from the country study team and WHO Global TB Programme.^6^

This first national TB patient cost survey in Mali aimed to baseline one of three End TB indicators in Mali’s national TB strategic plan. The study found that almost half of TB-affected households incurred costs higher than 20% of their annual household income, even though most TB services were offered at no cost. The WHO’s Global TB report 2021,^3^ based on 23 countries implementing national surveys, demonstrated that 47% (95% CI 33–61) of TB-affected households faced catastrophic costs due to TB. Nutrition supplementation and hospitalisation-related medical costs were the main drivers of high costs in Mali. Although many factors could contribute to this, a plausible explanation could be the lengthy delay between onset of symptoms and diagnosis (average 15.8 weeks, with over 90% of patients reporting a delay of >4 weeks). As a consequence, many patients are diagnosed when they are already sick and more nutritionally wasted, leading to longer hospitalisation.

**Table:** TB episode costs * borne by affected households in Mali, 2021 (in 2019 US$)

	TB patients (first-line)		Patients with drug-resistant TB		All TB patients	
		
	Mean	95% CI	Mean	95% CI	Mean	95% CI
Pre-diagnosis						
Medical	141	(125 to 157)	86.2	46.0 to 126.0)	140	(124 to 156)
Non-medical	24.1	(12.1 to 36.1)	7.17	(5.2 to 9.13)	23.6	(12 to 35.2)
Hours lost by patient x hourly wage	2.28	(2.01 to 2.56)	1.76	(1.05 to 2.46)	2.27	(2.0 to 2.54)
Post-diagnosis						
Medical	299	(205 to 393)	621	(0.0 to 1,378)	309	(215 to 403)
Travel	73.1	(56.7 to 89.6)	173	(94 to 252)	76.2	(59.1 to 93.3)
Accommodation	1.84	(−0.201 to 3.88)	0	(0.0 to 0.0)	1.78	(−0.196 to 3.76)
Food	27	(17.6 to 36.5)	88.1	(0.0 to 207)	28.9	(19 to 38.8)
Nutritional supplements	437	(295 to 579)	1,144	(354 to 1,933)	459	(316 to 602)
Hours lost by patient x hourly wage	35.6	(25.8 to 45.4)	467	(309 to 620)	48.9	(20.7 to 77.2)
Medical costs	440	(345 to 535)	707	(0.0 to 1,460)	449	(355 to 542)
Non-medical costs	563	(419 to 707)	1,412	(635 to 2,190)	589	(442 to 737)
Indirect costs (human capital approach)	35.6	(25.8 to 45.4)	467	(311 to 622)	48.9	(20.7 to 77.2)
Total episode costs per patient	1,039	(865 to 1,213)	2,586	(1,488 to 3,683)	1,087	(895 to 1,279)

* Indirect costs were measured using a valuation of time lost in care (human capital approach).

CI = confidence interval.

The contribution of indirect costs, computed through the valuation of time lost, to the overall costs of treatment was conservative. Sensitivity analysis, which calculated indirect costs using reported income loss (instead of using valuation of time lost), revealed that indirect costs were even higher, and would mean that 75% of households face catastrophic costs. The survey also revealed that households experienced unemployment, days of work lost and loss of food security. According to recent surveys,^7^–^12^ social protection policies are urgently needed to protect vulnerable households as they seek TB treatment to ensure higher treatment success rates. Key policy recommendations to mitigate these effects include implementing a more patient-centred model of care for TB patients, such as extending the reach of community health activities, improving linkages with nutrition support programmes and engaging more private facilities to reduce the time and costs related to repeat visits to the 83 facilities currently in the NTP network. Further collaboration between the Ministry of Health and local authorities is needed to improve the efficiency of decentralised services, while increased community engagement may enhance communication and improve accountability towards the End TB Goals. The main limitations of this cross-sectional design survey methodology have been reported previously^2^,^13^ and potential solutions suggested.^14^,^15^
